# Mechanisms of Action of the New Antibodies in Use in Multiple Myeloma

**DOI:** 10.3389/fonc.2021.684561

**Published:** 2021-07-08

**Authors:** Alessandra Romano, Paola Storti, Valentina Marchica, Grazia Scandura, Laura Notarfranchi, Luisa Craviotto, Francesco Di Raimondo, Nicola Giuliani

**Affiliations:** ^1^ Department of Surgery and Medical Specialties, University of Catania, Catania, Italy; ^2^ Department of Medicine and Surgery, University of Parma, Parma, Italy; ^3^ Azienda Ospedaliero-Universitaria di Parma, Parma, Italy; ^4^ U.O.C. Ematologia, A.O.U. Policlinico–San Marco, Catania, Italy

**Keywords:** monoclonal antibodies, multiple myeloma, CD38, SLAMF7, BCMA, antibody-drug conjugate, bispecific antibodies

## Abstract

Monoclonal antibodies (mAbs) directed against antigen-specific of multiple myeloma (MM) cells have Fc-dependent immune effector mechanisms, such as complement-dependent cytotoxicity (CDC), antibody-dependent cellular cytotoxicity (ADCC), and antibody-dependent cellular phagocytosis (ADCP), but the choice of the antigen is crucial for the development of effective immuno-therapy in MM. Recently new immunotherapeutic options in MM patients have been developed against different myeloma-related antigens as drug conjugate-antibody, bispecific T-cell engagers (BiTEs) and chimeric antigen receptor (CAR)-T cells. In this review, we will highlight the mechanism of action of immuno-therapy currently available in clinical practice to target CD38, SLAMF7, and BCMA, focusing on the biological role of the targets and on mechanisms of actions of the different immunotherapeutic approaches underlying their advantages and disadvantages with critical review of the literature data.

## Introduction

Multiple Myeloma (MM) is the second most frequent hematological neoplasm, due to uncontrolled proliferation of neoplastic plasma cells (PCs) in and out the bone marrow (BM), surrounded by a permissive and protective tumor microenvironment (TME) (1, 2). The cross talk between MM cells and their surrounding TME has been a major obstacle for the development of immunotherapy. However, thanks to increasing body of evidence about the molecular arms of MM/TME interaction and the introduction of multiple novel agents (3), median patient survival prolonged from 3 to 8–10 years (4). MM PCs are strictly dependent on BM microenvironment cells and they express different molecules on the surface as receptors and adhesion molecules that exploit the function of crosstalk and adhesion with the BM microenvironment (5). Some of these molecules, such as Cluster of Differentiation 38 (CD38), signaling lymphocyte activation molecule family member 7 (SLAMF7), and B cell maturation antigen (BCMA), are highly expressed by MM PCs characterizing them as good target for novel therapeutic strategies as monoclonal antibodies (6–8).

Monoclonal antibodies (mAbs) are a group of agents with immune-based mechanism of actions that in recent years have changed the management of newly diagnosed and relapsed/refractory MM (RRMM) (6, 9). Moreover, the development of a new generation of mAbs, including antibody-drug conjugates (ADCs) and bispecific antibodies (bsAbs) has the potential to additional improve the clinical outcome of MM patients (6, 10). Isotype dictates mAbs activity (11), and most anti-MM mAbs are IgG antibodies. The IgG subclass, allotype, and glycosylation pattern are the main factors involved in the interaction strength of the IgG-Fc domain with Fc engaging molecules, including the classical IgG-Fc receptors (FcγR), the neonatal Fc-receptor (FcRn), the Tripartite motif-containing protein 21 (TRIM21), the first component of the classical complement cascade (C1), the Fc-receptor-like receptors (FcRL4/5). The effector potential strength of the interaction between IgG mAbs and Fc engaging molecules will not be described, being out of scope of this manuscript. Several extensive and updated reviews are available about this topic (12, 13).

This review will describe main therapeutic targets in MM cells and the BM microenvironment and the mAbs in use in the anti-MM therapy focusing on their mechanism of actions and strategies to improve their efficacy.

## CD38

### Target Definition

Human Cyclic ADP ribose hydrolase, also known as CD38, is a 43.7 kDa type II transmembrane glycoprotein, encoded by CD38 gene located on chromosome 4 (14). E.L. Reinherz, S. Schlossman and colleagues, first identified this surface molecule in 1980 during their analysis of the human lymphocyte surface using mAbs in search of the T-cell receptor (15). Therefore, at the beginning, it was considered a marker of T cells; afterwards, it was exploited as a phenotypic marker to recognize and classify T and B leukemia (16).

### Physiological Expression and Function of CD38

This molecule is widely expressed in lymphoid and myeloid lineages (14, 17–19). Resting natural killer (NKs) cells and monocytes express it at low levels, as well as other cell types belonging to the hematopoietic lineage (**Table 1**): erythrocytes, platelets, and dendritic cells (DCs) (16, 20–22). Moreover, CD38 is expressed by different T cells subtypes T cell precursors as CD4^+^CD8^+^ double‐positive thymocytes (19). Within the circulating pool, CD38 is expressed by CD4^+^/CD45RA^+^ naive T cells as well as by subset of CD4^+^CD25^+^FoxP3^+^ regulatory T cells (Tregs) and by a subset of memory T cells (19, 23). CD38 is also a marker of activated T cells (19). Among CD8 T cells, CD38 is strongly expressed during chronic infection. CD38 is also expressed at high levels by peripheral blood mononuclear cells upon *in vitro* and *in vivo* activation (24). Subsequentially, CD38 expression is also modified during different stages of B cell differentiation. It is present at high levels on BM B cell precursors (immature or transitional) and is downregulated in mature B cells and is expressed at high level in terminally differentiated PC (19). Moreover, CD38 is expressed at high levels in a subset of B regulatory (Bregs) CD19^+^CD24^hi^ cells and on IL-10-producing plasmablast with regulatory functions, on the other hand memory B cell population show a low expression of CD38 (CD24^hi^CD38^lo^CD27^+^) (25). CD38 is also expressed on pathological cells such as chronic lymphocytic leukemia cells, where a presence of a major clone CD38^+^ positive is correlated with an unfavorable prognosis, and on MM cells (26). Analysis of CD38 distribution within MM bone niche revealed that only PCs express CD38 at high levels (27). Nevertheless, some studies demonstrated that CD38 expression is highly heterogeneous on MM cells, without a difference between newly diagnosed and relapsed/refractory MM patients (28). Moreover, in the MM bone microenvironment, CD38 decreases during osteoblasts (OBs) differentiation (29) and recently has been demonstrated that CD38 is expressed on the surface of early osteoclasts (OCs) progenitors but it is lost during *in-vitro* differentiation toward OCs (27).

**Table 1:** Expression of CD38, SLAMF7, and BCMA in cells circulating in peripheral blood.

Cell Type		CD38	SLAMF7	BCMA
***T-cells***	Precursor/double positive	+	+/−	−
	CD4^+^/CD45RA^+^ naive	+	+	−
	CD4^+^CD25^+^FoxP3^+^ regulatory	+	+	−
		*(subset)*		
	Memory	+	+	−
		*(subset)*		
	Activated CD8^+^	+	++	−
***B-cells***	Immature/transitional	+		−
	Mature	+/−	+	+
	Memory CD24^hi^CD27^+^	−/+	+	+/−
	Plasma cells	++	+	++
	CD19^+^CD24^hi^ regulatory	++	+	−
		*(subset)*		
	IL-10^+^ Plasmablast	+/−	+	−
		*(subset)*		
***NK-cells***	Progenitor	+	+	−
	Resting	+	+	−
	Activated	+	+	−
***Monocyte***		+	+	−
***Macrophage***		+	+	−
***Dendritic cells***	Immature	+/−	+/−	−
	Mature	+	+	−
***Erythrocytes***		+	−/+	−
***Platelets***		+	+/−	−

(+: positive; -: negative; +/-: weak positivity; -/+: mostly negative).

CD38 has a dual function of receptor and enzyme. As receptor, it regulates cellular adhesion, signal transduction, and calcium signaling. CD38 interacts with hyaluronic acid and the non-substrate ligand CD31, which is constitutively expressed by endothelial cells, leading to the activation of NF-kB, ZAP-70, and ERK1/2 pathways (26, 30). It has been generally known as a receptor despite a very short cytoplasmic tail that led to an inability to transduce the signal (6). Indeed, to act as a receptor, CD38 needs to be redirected to lineage-depended receptors of the cell membrane: BCR/CD19/CD21 in B cells, CD3/TCR in T cells, and CD16/CD61 in NK cells (26, 31).

The extracellular domain of CD38 acts as an ectoenzyme that, depending on the pH, is involved in the catabolism of nicotinamide adenine dinucleotide (NAD+) and nicotinamide adenine dinucleotide phosphate (NADP+) generating calcium signaling molecules, such as adenosine (ADO), that have immunosuppressive functions (32, 33).

All these data indicate multiple roles of CD38 in MM, becoming one of the most attractive antibody targets of the immunotherapeutic approaches to inhibit MM cell growth and survival and revert immunosuppression in MM patients.

### Monoclonal Antibodies Anti-CD38: *In Vitro* Molecular Rationale for Use

Several anti-CD38 antibodies have been developed in the last decade with different mechanisms of action. CD38-targeting antibodies such as daratumumab (DARA), MOR202, and isatuximab (ISA), have high single agent activity in heavily pretreated MM patients by pleiotropic mechanisms of actions (**Table 2**).

**Table 2:** Monoclonal antibodies against CD38 and SLAMF7 (Major clinical trials with published data).

Drug	Target	Manufacturer	Therapeutic format	Mechanism of action	Dose	Dose schedule	Clinical outcome in Monotherapy	Reference
Daratumumab	CD38	Janssen	naked mAb	ADCP, ADCC, CDC, cross-linking, immunomodulatory effect	16 mg/kg i.v.	Cycle 1–2 days 1, 8, 15, 22, cycles 3–6 days, cycle 7+ day 1	RRMM: ORR: 31.1%,	(34)
							Median PFS: 4.0 months	
							(95% CI, 2.8–5.6 months).	
							Median OS: 20.1 months	
							(95% CI, 16.6 months to NE)^1^	
Isatuximab	CD38	Sanofi-Aventis	naked mAb	ADCP, ADCC, CDC, direct apoptosis, adenosine inhibition	10 mg/kg i.v.	Cycle 1–4 days 1, 8, 15, 22, 29, cycle 4+ days 1, 15, cycle 18+ day 1	RRMM: ORR: 20%	(35)
							Median PFS: 4.6 months	
							Median OS: 18.7 months^2^	
Felzartamab (MOR202)	CD38	MorphoSys	naked mAb	ADCP, ADCC, CDC,	16 mg/kg i.v.	Days 1, 8, 15, and 22 of 28 days cycle	RRMM: ORR: 28% (+DEX)	(36)
Elotuzumab	SLAMF7	Bristol Myers Squibb/Celgene	naked mAb	ADCC, NK cells activation	0.5–20 mg/kg	Days 1, 15	RRMM: ORR: z10%	(37)

DARA is the first anti-CD38 mAb approved in MM therapy. It is a fully human immunoglobulin G1 kappa (IgG1ƙ) mAb (38) that binds two sequences of a unique CD38 epitope outside the catalytic domain (33, 39). It entered in clinical trial in 2015, for use as a monotherapy in the treatment of MM patients who had received at least three previous lines of therapy (40), now the use has been expanded to newly diagnosed MM.

ISA is a humanized IgG1k mAb, which binds to a specific 23-amino acid discontinuous epitope that include a part of the CD38 catalytic site and it has been selected due to its multiple mechanisms of actions (33, 41). ISA is now approved in combination with pomalidomide and dexamethasone, for the treatment of MM patients with who have received at least two prior therapies (42).

**Figure 1:**
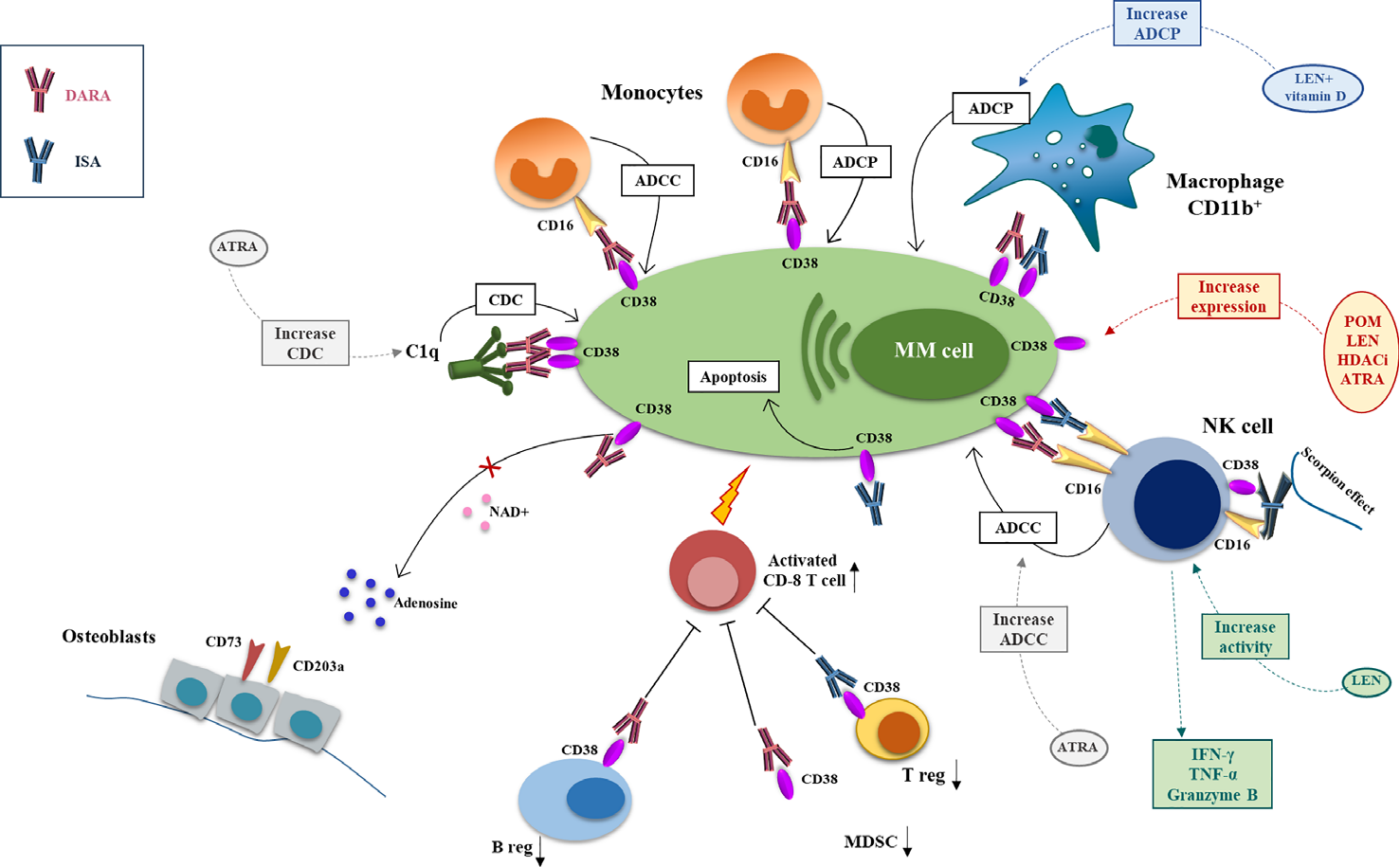
Mechanism of action and major drug combination of anti-CD38 mAbs, daratutmumab, and isatuximab. The anti-CD38 mAbs exert their antimyeloma activity through different mechanisms of actions that can be potentiate by different anti-MM drugs. CDC is activated by engagement of the C1q by DARA and initiates the classical complement cascade and the recognition of MM cells by phagocytic cells and the production of the anaphylatoxins. This mechanism can be increased by ATRA. ADCC involves NK cell and monocytes that through CD16 recognize the anti-CD38 mAbs on MM cell surface and activate the cytotoxic process. ISA can activate directly the NK cells through the scorpion effect. NK cell activity can be boosted by ATRA and LEN. ADCP is carried by CD16+ monocytes and CD11b+ macrophage; LEN+ vitamin D can enhance anti-CD38 mAbs-mediated macrophages phagocytic activity. ISA can also have a direct anti-MM effect inducing MM cell apoptosis. DARA has also an immunomodulatory function downregulating the immunosuppressor ADO, diminishing Breg and MDSCs and activating CD8+ T cells. ISA exerts its immunomodulating potential downregulating Treg (DARA, daratumumab; ISA, isatuximab; CDC, complement depend cytotoxicity; ADCC, antibody depend cytotoxicity; ADCP, antibody depend phagocytosis; ATRA, all-trans retinoic acid; LEN, lenalidomide).

Anti-CD38 antibodies exert their anti-MM activity by different mechanisms of action including classical FC-dependent immune effector mechanisms, namely the antibody-dependent cell-mediated cytotoxicity (ADCC) and antibody-dependent cellular phagocytosis (ADCP) and the complement-dependent cytotoxicity (CDC), and direct and immunomodulatory effects (43) (**Figure 1**).

#### Antibody-Dependent Cell-Mediated Cytotoxicity (ADCC)

Anti-CD38 antibodies can bind the Fc gamma receptors (FcγRs) (44) on the immune effector cells inducing the ADCC (40).

The binding with the Fc fragment of the anti-CD38 mAbs produces the intracellular phosphorylation of the tyrosine-based activating motifs of the FcγRs that leads in the lysis of MM cells (45). In particular the cell types FcγRs-expressing that are mainly involved in the ADCC-anti-CD38 mAbs mediated are NK cells which express CD32 and CD16, monocytes expressing CD16 and macrophages CD64^+^ (45).

NK cells are probably the main mediator of ADCC by mAbs. *In vitro* and *ex-vivo* data demonstrated that, DARA, by its binding with CD16, induces NK cells activation through the induction of STAT1 phosphorylation and activation of NF-kB p65 (46). Activated NK produced pro-inflammatory cytokines, as interferon gamma (IFN-γ) and tumor necrosis factor alpha (TNF-α) that lead to the recruitment of immune cells and MM cells killing (45, 46). Recent *ex vivo* data report the important role of the BM adaptive NK cell, characterized by a lower expression of CD38 and high expression of NKG2C, an activating NK receptor, in the response to DARA treatment of newly diagnosed MM patients (47). In particular, this NK subset sorted from BM of MM patients have higher ADCC capacity to kill MM cell coated with DARA compared to the conventional NK cell and adaptive NK cell frequencies is correlated with DARA response *ex vivo* (47).

Moreover, DARA could also enhance CD38^+^ NK cell apoptosis through a fratricide NK-to-NK ADCC without the involvement of tumor cells (46). This mechanism could be the basis of the rapid depletion of NK cell in MM patients after DARA treatment. Finally, also CD14^+^CD16^+^ monocytes can induce ADCC against MM cell coated by DARA (43, 48, 49).

On the other hand, *in vitro* data support that ISA induces ADCC by NK cells more efficiently against MM cells with higher density of CD38 on the surface, leading to the production of INF-γ and TNF-α (50). Moreover, it is hypothesized that ISA can directly activate NK cells through the cross-link of CD38 and CD16 on their surface (the scorpion effect) and activated NK cells can kill CD38^low^ and CD38^−^ target cells (50–52). Finally, Moreno et al. suggest that the depletion of NK cells after ISA treatment could be imputed to an exhaustion of these cells due to the higher ISA-mediated activation, rather than a fratricide mechanism (51).

#### Antibody-Dependent Cellular Phagocytosis (ADCP)

Phagocytosis contributes to the anti-MM activity of the anti-CD38 mAbs, as well (43). *In vitro* studies have demonstrated that DARA-coated MM cells are rapidly engulfed by tumor-associated macrophages (53). Recently, it has been demonstrated in an *ex-vivo* assay that the CD16^+^ subset of monocytes is essential in DARA MM cells-killing activity and the inhibition of the anti-phagocytic signal CD47-SIRPα significantly improves the DARA effect mediated by CD16^+^ monocytes (49).

On the other hand, Moreno et al. demonstrated that *in vitro* ISA triggers ADCP by CD11b^+^ macrophages only on MM cells that present a high level of CD38 molecules on the surface and the ability of ISA to induces ADPC also in NOD/scid/γc^−/−^ (NSG) mice (51).

#### Complement-Dependent Cytotoxicity (CDC)

The Fc tail of the anti-CD38 mAbs engage the C1q molecule and initiates the classical complement cascade, leading the deposition of C3b on MM cell surface inducing the CDC, the recognition by phagocytic cells and the production of the anaphylatoxins C3a and C5a (40, 45). This effect could be imputed to a mechanism recently described by different groups on anti-CD20 mAbs: the establishments of non-covalent interactions between the mAbs Fc tails resulting in the formation of antigen dimerization that adjuvate the constitution of antibody hexamers after antigen binding on cells that recruit and activated C1 (54–56). DARA is the most effective inducer of CDC, while ISA can induce CDC only in a few MM samples with high expression of CD38 on PCs (51). The *in vitro* CDC induction by both DARA and ISA is reduced in presence of high level of inhibitory complement regulatory proteins CD59 and CD55 on MM cells (28, 50).

#### Immunomodulatory Effects

As CD38 is expressed on several immune cells, anti-CD38 mAbs have also immunomodulatory effects. DARA treatment reduced CD19^+^C24^+^CD38^+^ Bregs in MM patients and *in vitro* generated MDSCs (CD11b^+^CD14^−^HLA-DR^−^CD33^+^CD15^+^CD38^+^) causing a modification of the antitumor response (57). Moreover, DARA induces CD4^+^ and CD8^+^ T cells expansion in MM patients and in particular the effector memory CD8^+^ T cells concomitant with a decrease of naïve T cells subset (57). Indeed, the reduction of immunosuppressive cells could lead to an increase in T-cell numbers, T-cell clonality, as well as T-cell activity contain higher levels of granzyme B after exposure to DARA (57, 58).

Like DARA, ISA reduces T regulatory cells (Tregs) and blocks the production of immune inhibitory cytokines like interleukin (IL)-10 (59). Recently it has been demonstrated that ISA also depletes CD38^hi^ B lymphocyte precursors and NK cells (51).

Finally, DARA treatment possibly modulates the enzymatic activity of CD38 by reducing the ADO levels. The axis CD38/CD203a/CD73 converts NAD^+^ to ADO: NAD+ reduction leads to the development of exhausted T cells and adenosine has an immunosuppressive effect on NK and CD8^+^ cells (60, 61). Van de Donk et al. showed that DARA reduces CD38 cyclase activity, increasing NAD^+^ levels and decreasing ADO levels (58). Indeed, targeting CD38 with anti-CD38 mAbs could restore the immune functions.

#### Direct Effects

ISA was selected initially based on its *in vitro* ability to directly induce MM cell death independently of effector cells and independently of Fc fragment binding to FcRs by binding the CD38 activating the classical caspase, lysosome death pathways, lysosomal membrane permeabilization, and cathepsin hydrolase release (62). Moreover, it is reported that ISA could induce reactive oxygen species production and promote to MM cell death (62). In contrast with these data, a recent paper reported no direct killing activity on ISA on MM cell *in vitro* (51).

In contrast, DARA did not show a direct killing effect on MM cells (43).

#### Mechanisms to Potentiate the Effects of Anti-CD38 mAbs in MM

Several pre-clinical studies indicate that the effects of anti-CD38 may be potentiated with other drugs and compounds. A synergistic effect between DARA and lenalidomide (LEN) in the induction of ADCC cytotoxicity has been previously demonstrated (63). Indeed, it is known that LEN stimulates NK cell increasing their production of IFN-γ, TNF-α, and granzyme B (64). Interestingly, an up-regulation of DARA-dependent ADCC was described in peripheral blood mononucleated cells (PBMCs) isolated from MM patients during or just after LEN treatment, thus further supporting the potential benefits from this combination (63).

Other studies showed that LEN enhances DARA-induced MM cell lysis by an increased frequency of CD3−CD56+ NK cells, with no alterations of T cell and monocyte compartments, even in patients refractory to LEN (65). Consistently, data obtained in humanized mice engrafted with MM cells from LEN refractory patients confirmed the capacity of LEN to potentiate the DARA effect (65). Accordingly, Van der Veer et al. (66) showed that the synergism between DARA and LEN/bortezomib treatment was more prominent in CD138^+^ CD38^+^ cells of MM patients refractory to LEN (66).

More recently, it has been also suggested that also vitamin D can potentiate the synergism between LEN and anti-CD38 mAbs combination mediated by the increase of the ADCP (67) due to LEN ability to induce CYP27B1 expression in macrophages (68). DARA-LEN synergism could be also due to the decrease of the frequency of inhibitory T cell populations induced by LEN (69). Indeed, it has been demonstrated that LEN up-regulates CD38 expression on Tregs and increases the fraction of CD38-high Tregs sensitizing this population to the anti-CD38, ISA (59).

Our group previously demonstrated that LEN and pomalidomide (POM) up-regulate CD38 expression by MM cells (70). This finding was recently confirmed by others (71): showing that the activity of DARA in combination with LEN was correlated by the increased CD38 surface expression by MM cells but not by NK cells by LEN (71). Lastly, Jiang et al. (62) showed that POM, enhances anti-CD38 mAbs effect both by the direct killing of MM cells, and by the indirect cytotoxicity effect (62). Interestingly it has been also reported that POM synergized with ISA in CD38-high MM cells with mutated p53 (62) supporting the use of POM and ISA combination in this type of high-risk MM patients.

Panobinostat is a pan-HDACi able to increase the expression of CD38 by PCs but not T cells (72) and consequently to potentiate the effect of DARA (72). Ricolinostat also increases CD38 expression on the surface of MM cells and it augments the ADCC by DARA against MM cell lines but not CDC effect (73).

Other drugs could be also used to increase the efficacy of anti-CD38 mAbs through the modulation of CD38 expression by MM cells and/or the effector cells. Different studies showed that agents such all-trans-Retinoic acid (ATRA) can be used to improve the effect of DARA and to overcome its resistance by increasing the expression of CD38 in MM cell (28). Nijhof et al. showed that treatment with ATRA significantly increased the expression of CD38 enhancing DARA-induced ADCC and CDC (28). The mechanism involving the modulation of CD38 by ATRA can be explained by the presence of a retinoic acid responsive element located in the first intron of the CD38 gene (74). Interestingly, treatment with ATRA also reduced the expression of CD55 and CD59 in MM cells (75). These studies clearly provide the rationale to design clinical trial with ATRA and DARA in refractory MM patients.

#### Bispecific Antibodies Against CD38

An antibody containing two different antigen-binding sites within one molecule is known as a bispecific antibody (BsAb). In particular, Bispecific T-cell engaging (BiTE) antibodies are a new class of drugs that can bind both a specific antigen on the surface of the tumor cells and the CD3e chain on T cells (76). BsAbs that target CD38 are in developing the last years and some of them are under evaluation in Phase I studies.

AMG424 is a novel CD38/CD3 BiTE, and recently it has been reported that AMG 424 can kill cancer cells expressing high and low levels of CD38 *in vitro* and increases T-cell proliferation, but with attenuated cytokine release (77). However, since CD38 is expressed in normal immune cells and non-hematopoietic tissues, it is associated with off-target toxicity (77). A phase 1 first-in-human trial (NCT03445663) of the drug in patients with R/R MM started in July 2018.

This year, it has been published a new BiTe against CD38: Bi38-3. Bi38-3 is made of two single-chain variable fragments anti-human CD38 and CD3e; it activates T-cell-mediated lysis of CD38^+^ MM cells *in vitro*, *ex vivo*, and *in vivo*. Moreover, it has been reported that it has no toxicity on B, T, and NK cells *in vitro* (78). Furthermore, Bi38-3 triggers the killing of MM cells from resistant patients and, since it recognizes a specific epitope on the Fc region of CD38, could be efficient also in patients after daratumumab therapy (78).

## SLAMF7/CS1/CD319

### Target Definition

The signaling lymphocyte SLAMF7, also known as CRACC or CD319 is encoded by SLAMF7 gene present on chromosome 1 at locus 1q23-24 (79, 80), is a 66kDa glycoprotein member of the SLAM superfamily. The SLAM family include several related CD2 subset of the immunoglobulin superfamily of receptors expressed on the surface of a wide variety of hematopoietic cells, including CD150, CD48, CD244, CD229, CD84 NK-T-B-antigen (NTB-A), also known as SF2000 in human or Ly108 in mouse, CD352, CD319, B lymphocyte activator macrophage expressed (BLAME, Slamf8), and SF2001 (CD84H, Slamf9) (79).

Like most SLAM receptors, SLAMF7/CS1 is a self-ligand, which exert activating or inhibitory influences on cells of the immune system depending on cellular context and the availability of effector proteins (81, 82).

SLAMF7 contains a membrane proximal C-type Ig fold and a membrane distal V-type Ig, a cytoplasmic region including two immunoreceptor tyrosine-based switch motifs (ITSM). The phosphorylation of tyrosine-based motifs in SLAMF7 induces downstream molecules activation including PLCγ1, PLCγ2, and PI3K kinases, regulating a variety of cell functions. SLAM receptors triggered by homo- or heterotypic cell-cell interactions control the activation and differentiation of a wide variety of immune cells and the interplay between innate and adaptive immune response. Downstream signaling is mediated by recruitment of small cytoplasmic adapter proteins, namely SH2D1A/SAP and/or SH2D1B/EAT-2. In humans, SLAMF7/CS1 has two splice variants, constitutively expressed on NK cells, namely a long-form CS1-L and a short form CS1-S, which lacks an ITSM motif required for NK cells activation described above (83). CS1L mediates NK cell activation through a SH2D1B/EAT-2 dependent, SH2D1A/SAP-independent extracellular signal- regulated ERK-mediated pathway. Thus, SLAMF7 can act as an activator if it can bind EAT-2, otherwise it is an inhibitor of downstream signaling (82).

### Physiological Expression and Function of SLAMF7

SLAMF7, first described in NK cells (84, 85) and macrophages, is involved in numerous functions, including PCs survival, cell adhesion, NK cell– and CD8 T cell–mediated cytotoxicity (79). Several other hematopoietic cells express SLAMF7, including myeloid cells, activated T cells, most B cells, including antibody-producing PCs (81).

In NK cells, SLAMF7 is usually a positive regulator of NK cell activation, as consequence of the binding with the SAP family adaptor Ewing’s sarcoma-associated transcript 2 (EAT-2) *via* phosphorylated tyrosine 281 (Y281) in its cytoplasmic segment, thereby triggering activating signals involving phospholipase C-γ (PLC-γ) (86, 87) to induce polarization of cytotoxic granules (86). In the absence of EAT-2, or in excess of another adapter protein SAP (87), SLAMF7 recruits SHIP-1 and mediates inhibitory effects, as found in NK cells derived from EAT-2-deficient mice, and in normal activated T cells (81) and MM PCs (82), which lack EAT-2.

### Expression and Function of SLAMF7/CS1 in Multiple Myeloma

Primary myeloma cells and human myeloma cell lines express higher levels of SLAMF7 than the normal or reactive counterpart, as consequence of genetic derangements (**Table 1**). In particular, cancer cells carrying the translocation t(4; 14) seem to have a greater expression of SLAMF7 and the *in vitro* inhibition of the expression of SLAMF7 in these cells is able to reduce the formation of colonies and to induce apoptosis and arrest of cells in G1, thus indicating an important role of this receptor in the proliferation of MM cells (88). The promoter region of SLAMF7/CS1 can bind the identity marker of PCs Blimp-1 (B-lymphocyte-induced maturation protein-1), which is dysregulated in MM, to enhance SLAMF7/CS1 transcription (89). This finding could explain why increased expression of SLAMF7 has been reported also in other B-cell disorders, like chronic lymphocytic leukemia and diffuse large B cell lymphoma (90). High levels of soluble SLAMF7 (sCS1) (91, 92) and increased mRNA of SLAMF7 in purified PCs have been documented in patients affected by monoclonal gammopathies in the entire spectrum, from monoclonal gammopathy of unknown significance (MGUS) through smoldering-, active-, and relapsed-refractory MM relapsed patients (93) and in autoimmune diseases, like systemic lupus erythematosus (94), or systemic infections in response to IFN-⍺ stimulation (80).

In 199 newly diagnosed MM patients, the amount of sCS1 was positively associated to active MM and not appreciated in healthy or stage I MM patients (95). Increased sCS1 was associated to most aggressive presentation, in both newly diagnosed and relapsed MM-patients, associated to lower probability to achieve deep response and reduced progression free survival, even if it could not be shown as an emerging independent prognostic factor (91).

### Monoclonal Antibodies Anti-SLAMF7: *In Vitro* Molecular Rationale for Use of Elotuzumab

Elotuzumab is a humanized IgG1 mAb directed selectively against SLAMF7, unable to induce direct or complement-mediated lysis of MM cells, as shown *in vitro* and by absence of clinical activity as single agent (82, 96) (**Table 2**). In MM cells, high SLAMF7 expression is not able to induce neither proliferation nor apoptosis, due to lack of both EAT-2 (required to activate downstream signaling) and SHIP-2 (required to inhibit of downstream signaling) (82). Targeting SLAMF7/CS1 *in vitro* inhibited cell viability of human MM cell lines co-cultured with bone-marrow stromal cells (BMSCs) in a dose-dependent fashion, overcoming the stimulatory and protective effects of microenviroment on MM growth and survival (95), implying that its efficacy occurred through an indirect mechanism.

Elotuzumab can induce SLAMF7 expression in NK cells and acting as self-ligand can amplify its molecular effect (97). Favoring the homotypic SLAMF7-SLAMF7 interaction between NK and MM cells, elotuzumab further promotes natural cytotoxicity in a CD16-independent manner (85). Indeed, an Fc mutant form of Elotuzumab, unable to bind CD16, could promote cytotoxicity of SLAMF7+ target cells by NK cells derived from healthy donors, in particular when these cells were previously exposed to IL2. Therefore, it is likely that additional effects of Elotuzumab on immune system are based on involvement of other SLAMF7+ cells such as CD8+ lymphocytes (98), dendritic cells, activated monocytes, and dendritic plasmacytoid cells (99), as shown by reduced efficacy in CD8+ T- cells-depleted mice (98). Elotuzumab may inhibit the plasmocytoid dendritic cells that play an important role within the microenvironment that support MM cells growth and survival (99).

### *In Vivo* Effects of Elotuzumab in MM and Strategies to Improve Clinical Efficacy of Immunotherapy Addressed Against SLAMF7

**Figure 2:**
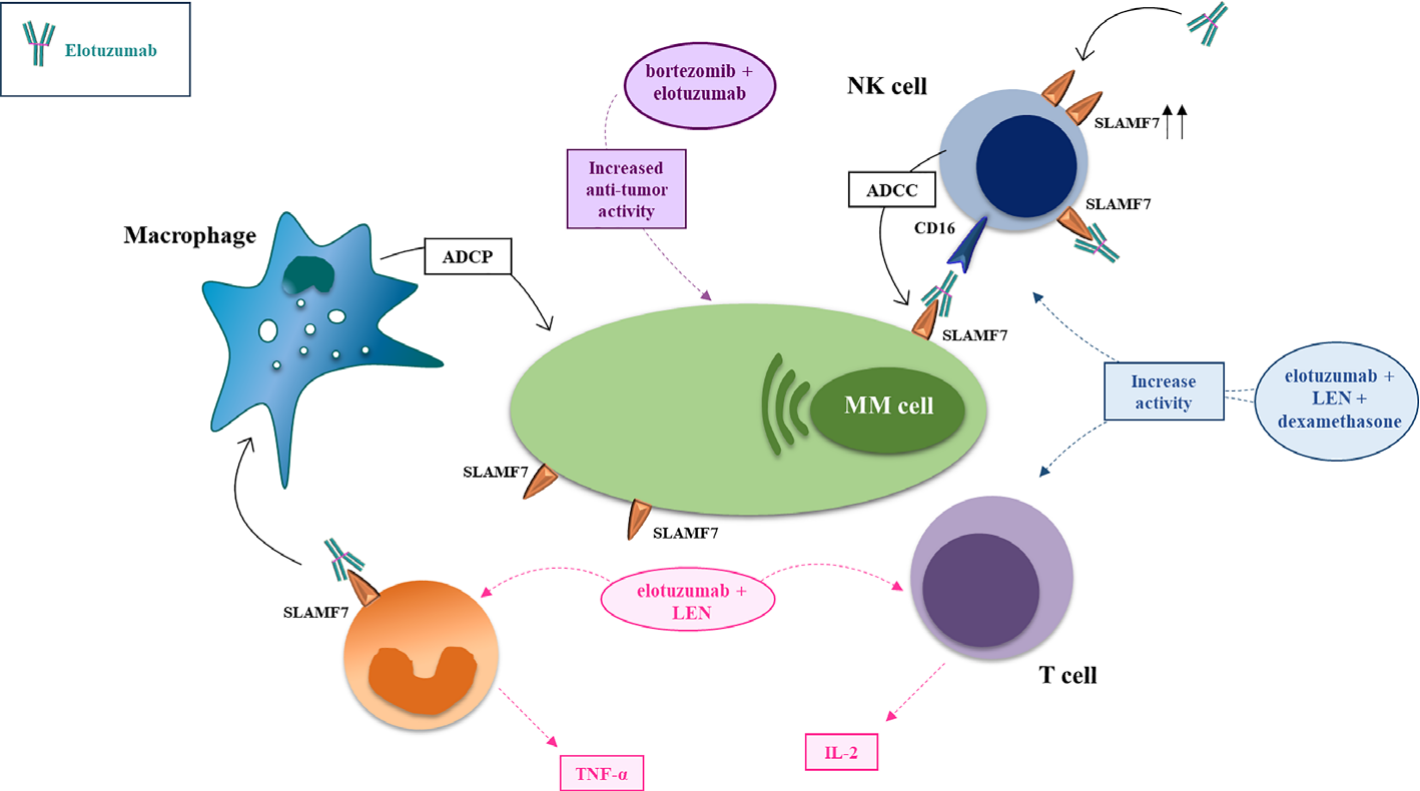
Mechanism of action and major drug combination of the anti-SLAMF7 mAb elotuzumab. The anti-SLAMF7 mAb elotuzumab exerts anti-MM effects *via* several indirect mechanisms: (i) promoting macrophage-mediated antibody-dependent cellular phagocytosis (ADCP) engaging co-stimulatory signaling to enhance ADCP in macrophages expressing both SLAMF7 and EAT-2; (ii) facilitating NK cell-mediated antibody-dependent cellular cytotoxicity (ADCC) of myeloma cells through Fc-dependent interactions with CD16 (FcγRIIIA); (iii) enhancing co-stimulatory signaling in NK cells, thereby potentiating natural cytotoxicity of myeloma cells, *via* simultaneous engagement of ITAM-linked activating receptors on NK cells (e.g. NKp46 or CD16) with ligands on myeloma cells; (iv) tagging myeloma cells for cell recognition; (v) elimination of immunosuppressive CD8+CD28−CD57+ Tregs which overexpress SLAMF7. In combination with proteasome inhibitors (e.g. bortezomib, carfilzomib) or immunomodulators (e.g. lenalidomide, pomalidomide), elotuzumab enhances anti-tumor effects *via* activation of T-cells and NK-cells.

*In vivo*, most activity of elotuzumab can be attributed to NK-cells engagement *via* two main mechanisms. First, ADCC triggering, *via* engagement of FCγRIIIa/CD16a (93, 95). Second, a direct binding of elotuzumab’s Fab domain with the SLAMF7 receptor enhances EAT-2 recruitment to promote NK cells activation (81, 82, 84, 85, 87, 97, 100, 101). For this reason, differently from daratumumab, another mAb used in the same setting of relapsed/refractory (RRMM) patients, elotuzumab does not affect viability of NK cells (102), explaining the increasing interest for this molecule in the emerging adoptive CAR NK-cell therapies (103) (**Figure 2**).

To increase the cytotoxic effect of elotuzumab several strategies have been explored. In general, the combination with drugs able to induce SLAMF7 expression, recruit NK-cells and promoting ADCC is been extensively evalauted and it is highly recommended. In the pivotal studies, Van Rhee et al. treated SCID-human xenograft mice and documented that antitumor activity was enhanced if the MM cells were pretreated with bortezomib, even if pretreatment with bortezomib did not affect SLAMF7 expression (96).

In mice, the combination of lenalidomide and elotuzumab was very effective in reducing tumor volume and increasing the infiltration of NK cells into the tumor microenvironment, an effect enhanced by IL-2 secreted by T cells and TNF-alpha produced by monocytes (101) and macrophages (104). According to observation that Elotuzumab is able to reduce tumor burden and prolong survival in a MM model with SCID-beige mouse lacking B-, T-cells and with a reduced NK function, a further mechanism of action has been recently proposed. Elotuzumab could recruit monocytes, promote the infiltration of M1-polarized tumor-associated macrophages with enhanced ADCP of MM cells through engagement of the Fc*γ* receptor (104).

Immunomodulatory drugs (IMiDs) look like the ideal backbone to combine with Elotuzumab for their direct and indirect effects on both T- and NK-cells function (64, 105, 106), as shown in the relapsed/refractory setting by trials ELOQUENT-2, which tested efficacy and safety of elotuzumab combined to lenalidomide and dexamethasone (107, 108) and ELOQUENT-3 study, which tested efficacy and safety of elotuzumab combined to pomalidomide and dexamethasone (109). Unfortunately, the ELOQUENT-1 trial, which evaluated elotuzumab combined to lenalidomide and dexamethasone in the setting of newly diagnosed, transplantation-ineligible MM patients, failed to demonstrate additive clinical activity of elotuzumab (110).

Elotuzumab can be combined with other mAbs to increase the activity of effector cells, like the checkpoint inhibitor pembrolizumab (111) to promote tumor-infiltrating NK and CD8+ T-cell activation, intratumoral cytokine and chemokine release (98).

Elotuzumab has ben used to arm an anti-CD3 (OKT3) antibody to develop a bispecific antibody-armed activated T cell to induce adaptive cellular and humoral immune responses in MM patients, to mediate MM cytotoxicity independently from major histocompatibility complex. *Ex vivo* arming unarmed activated T cells avoids the need to administer large quantities (mg/kg) of bispecific antibody to reduce adverse events, like cytokine release syndrome. This strategy utilizes humoral antibody targeting by ATC. Secretion of Th1 cytokines upon binding of the effector cells to the myeloma cells not only augments tumoricidal activity directed at the malignant B cells, but may increase local cytokine and chemokine secretion that leads to shifting the tumor microenvironment to recruit endogenous immune effectors and induce an endogenous immune response (112). The targeting domain derived from elotuzumab has been used to develop T cells expressing an SLAMF7 CAR, with promising activity in preclinical models *in vitro* and *in vivo*, leading to ongoing phase 1/2a clinical trials CARAMBA and MELANI-01 (113).

Finally, a SLAMF7-targeted mAb ahs been conjugated with a payload drug (e.g. DM1, DM4, SN38, MMAE, MMAF) through a linker (e.g. SMCC, SPDB, MC-Vc-PAB). Azintuxizumab vedotin (ABBV-838) was the first-in-class antibody-drug conjugate (ADC) in which a SLAMF7-targeted mAb was linked to monomethyl auristatin E (MMAE) *via* a cathepsin B-cleavable peptide linker. Two phase I clinical trials have been started but in June 2017 AbbVie decided to terminate the phase-I/Ib trial NCT02462525 for insufficient clinical activity (114), with only 10% of overall response rate (115).

## The BAFF-APRIL-BCMA System

### Target Definition

The persistence of normal and neoplastic plasma cells (PCs) depends on survival factors provided in the bone marrow as consequence of direct contact to mesenchymal stromal cells (116) or the B-Cell Maturation Antigen (*BCMA*) triggering, induced by its two ligands, namely, B-cell-Activating Factor (*BAFF*; *BLys* and *CD257*) (117) and A PRoliferation-Inducing Ligand (*APRIL; CD256*), that are respectively produced mainly by macrophages (118) and osteoclasts (119, 120).

*BCMA* (also referred as TNFRSF17, CD269) is a transmembrane glycoprotein belonging to the tumor necrosis factor superfamily, selectively induced during B-cell differentiation into plasmablasts and bone marrow PCs (121), neoplastic PCs (120, 122), while it is nearly absent on naive and memory B cells (121, 122) CD34 stem cells, and other normal tissue cells (123) (**Table 1**). In normal and neoplastic mouse plasma cells, and in the human MM cell line MM1.s, the BCMA expression is under control of the master plasma cell gene IRF4 (124, 125), even if the post-translational regulation of BCMA can be largely compensated for reduced transcription, by mechanisms still under investigation (124).

Following stimulation with APRIL or BAFF, BCMA becomes a trimer, eliciting a signaling cascade involved in the activation of MAP kinases and the induction of anti-apoptotic proteins, such as Bcl-2, Bcl-XL, and the antiapoptotic protein myeloid cell leukemia 1 (MCL-1) (126).

### The BAFF-APRIL-BCMA System Regulates Plasma Cells Homeostasis

*BAFF* is required for homeostasis and maintaining normal B-cell development, and survival of malignant B- and PCs, by increasing the levels of the pro-survival molecules B cell lymphoma 2 (Bcl-2) and Bcl-x and by decreasing the levels of the proapoptotic molecule Bcl-2-homologous antagonist/killer (Bak) (127, 128). BCMA^ko^ mice have shorter survival of long-lived bone marrow PCs compared to wild-type controls while maintaining a normal phenotype (129). BAFF binds mainly the BAFF Receptor (BAFF-R), which triggers naïve B cell survival and maturation. During B-cell development BAFF-R is first expressed on immature B cells with the highest expression levels on transitional and mature B cells and decreased levels on germinal center B cells, while BCMA and TACI (Transmembrane activator and CAML interactor) are expressed in a more restricted manner and support the survival of PCs. BAFF/BCMA binding activates NF-kB and the MAPK8/JNK signaling pathways, to sustain long-term humoral immunity, survival, and proliferation to regulate B cell antibody responses, isotype switching, and homeostasis (130). While BAFF is required for B cell homeostasis, the excessive production of BAFF is detrimental to the host. Transgenic mice overexpressing BAFF suffer from increased production of autoantibodies and symptoms of autoimmune diseases (130–132).

*APRIL* binds to BCMA with higher affinity interaction than BAFF to prevent activation of the endoplasmic reticulum (ER)-associated Casp12 contributing to maintenance of long-lived PCs in the niche (116). APRIL can also bind heparin sulfate proteoglycans to potentiate TACI and BCMA activation through its multimerization (133, 134), but the underlying molecular mechanisms are still largely unknown.

BAFF and APRIL are equally potent in inducing bone marrow plasma cell survival. *TACI* mediates the BAFF- and APRIL-induced generation of PCs and T cell-independent immunoglobulin isotype switching and secretion, whereas the function of *BCMA* is restricted to the maintenance of PCs and antigen presentation by B cells, through the activation of AKT, MAPK, and *via* NF-kB (120).

BCMA is shed from the surface of PCs *via* γ-secretase–mediated cleavage, with consequent releases of soluble BCMA (sBCMA). sBCMA acts as a decoy neutralizing APRIL (135) and sequesters B-cell activating factor BAFF (136), thereby preventing it from performing its signaling to stimulate normal B-cell and plasma cell development, resulting in reduced polyclonal antibody levels (136).

### The BAFF-APRIL-BCMA System in Multiple Myeloma

BCMA is detectable in malignant PCs throughout the duration of the disease, with progressive increased expression from monoclonal gammopathy of uncertain significance (MGUS) to smoldering myeloma to active MM, with the highest levels correlated to the worst prognosis (137). In MM patients, BCMA mRNA is upregulated in PCs, and in CD138− progenitor cells responsible for recurrences (138). Conversely, a downregulation of BCMA reduces the viability and formation of myeloma colonies (120). *In vivo*, BCMA-overexpressing tumors increased neo-angiogenesis and transcription of genes crucial for osteoclast activation, adhesion, and angiogenesis/metastasis, as well as genes mediating immune inhibition including programmed death ligand 1 (PDL1), transforming growth factor β (TGF- β), and interleukin 10 (IL-10) to orchestrate the complex interplay between myeloma and microenvironment cells (120).

Soluble BCMA (sBCMA) is higher in supernatants of mononuclear cell cultures of MM-BM than the marrow of healthy subjects (135), progressively increased from healthy, MGUS, and active MM, and higher in patients with progressive disease, associated to reduced overall survival (139). In mice, sBCMA levels correlated with the change in tumor volume in response to melphalan or cyclophosphamide with bortezomib (139). *In vivo*, sBCMA correlated with the percentage of bone marrow PCs, even in patients with non-secretory myeloma and with the depth of response to treatment. Patients with levels above the median had shorter progression free survival and overall survival (140). Normalization of sBCMA during any treatment was predictor of increased overall response rate, overall survival (141), and achievement of complete response (141). Currently, sBCMA (139, 140) and sTACI are investigated as novel biomarker of disease activity in B-cell disorders with prognostic value (142), with two main advantages: independence from renal kidney and shorter half-life (24–36 h) than monoclonal components IgG (21 days) and IgA (7 days), or free light chains (140, 141).

Based on the above-mentioned circuitry, BCMA has been recently evaluated as highly selective antigen for neoplastic PCs, representing that tumor associated antigen ideal for the development of target therapy (143). Moreover, due to lack of expression on B-cell precursors, a rapid recovery of cell B immunity could be expected upon discontinuation of anti BCMA treatment.

### How to Target the BAFF-APRIL-BCMA System in Multiple Myeloma: Tabalumab

*Tabalumab* (LY 2127399) is an-anti BAFF human mAb developed by Eli Lilly and Company, designed for the treatment of autoimmune diseases and B cell malignancies (144). In MM, two phase II studies, conducted in US and Japanese cohorts, failed to show any clinical improvement in progression free survival (145–147), probably for high BAFF concentrations in RRMM patients or the induction of compensatory pathways *via* APRIL/BCMA engagement (148–151), suggesting the need to combine tabalumab with anti‐APRIL antibody or TACI‐Fc fusion protein, a potent inhibitor of both BAFF and APRIL, to augment clinical efficacy in RRMM.

### How to Target BCMA in Multiple Myeloma: The Antibodies Drug Conjugates

Currently, the development of immunotherapy against BCMA is directed towards three approaches: antibodies drug conjugates (ADCs), the bispecific antibodies, and the CAR-T cells, that have been recently described in a comprehensive review (**Tables 3** and **4**) (163).

**Table 3:** BsAbs against BCMA in clinical development (Major clinical trials with published data).

Drug	Target	Manufacturer	Therapeutic format and Mechanism of action	Dose	Dose schedule	Clinical outcome in Monotherapy	Reference
AMG420 (former BI 836909)	BCMA/CD3ϵ	Boehringer Ingelheim/Amgen	Bispecific single-chain variable fragment with hexahistidine tag antibody	0.2–800 µg/day I.V.	4 weeks of continuous I.V. infusion over a 6-weeks treatment cycle	RRMM, ORR 31%,	(152)
						70% at 400 μg/d (N = 7)	
Pavurutamab (AMG701)	BCMA/CD3ϵ	Amgen	Bispecific single-chain variable fragment with hexahistidine tag antibody	Phase I dose-escalation study	4 weeks of continuous I.V. infusion over a 6-weeks treatment cycle	RRMM, ORR 26%,	(153)
						83% at 18 mg dose (N = 6)	
CC-93269 (former BCMA-TCB2/EM-901)	BCMA/CD3 (Dual BCMA binding site)	Celgene	Asymmetric two-arm IgG1-based human bispecific T-cell engaging antibody. In EM 901the heterodimeric Fc region has intact FCRn binding site	Phase I dose-escalation study	I.V. @ on days 1, 8, 15, and 22 of cycles 1 to 3, on days 1 and 15 of cycles 4 to 6, and on day 1 of cycle 7	RRMM, ORR 43%,	(154)
						89% at 10 mg dose (N = 9)	
TNB-383B	BCMA/CD3 (Dual BCMA binding site)	TeneoBio and Abbvie	T-cell engaging bispecific antibody, with unique selective activating anti-CD3 moiety, two heavy-chain-only anti-BCMA moieties for a 2:1 tumor associated antigen to CD3 stoichiometry, with an IgG4 silenced backbone to reduce nonspecific T-cell activation	Phase I dose-escalation study	1–2 h I.V. infusions every 3 weeks	RRMM, ORR 47%,	(155)
						80% at 40–60 mg doses (N = 15)	
Elranatamab (PF-06863135)	BCMA/CD3	Pfizer Alexo Therapeutics Kodiak Sciences	Fully human IgG CD3 bispecific molecule, with IgG2A backbone	Phase I dose-escalation study,	Weekly subcutaneous	RRMM, ORR 53%,	(156)
				80–360 μg/kg (SC)		80% at 215–1,000 µg/kg mg doses (N = 20)	
				0.1–50 μg/kg (I.V.)			
Teclistamab (JNJ-64007957)	BCMA/CD3	Janssen Pharmaceutical Companies	DuoBody. Bispecific IgG1 molecule generated by controlled Fab-arm exchange of two separated mAbs	80–3,000 μg/kg (SC)	Weekly I.V./SC	RRMM ORR 64%,	(157)
				0.3–720 μg/kg (I.V.)			
REGN5458	BCMA/CD3	Regeneron and Sanofi	BCMA x CD3 bispecific antibody	Phase I dose-escalation study	Weekly I.V. ×16, then every 2 weeks	RRMM ORR 39%,	(158)
				3–96 mg		63% at 96 mg dose (N = 8)	

**Table 4:** ADCs against BCMA in clinical development.

Drug	Target	Manufacturer	Therapeutic format and Mechanism of action	Dose	Dose schedule	Clinical outcome in Monotherapy	Reference
Belantamab (former GSK2857916)	BCMA	GSK	mAb: afucosylated IgG1 humanized αBCMA linker: non-cleavable, protease resistant payload: MMAF	3.4 mg/kg	30–60 min I.V. infusions every 3 weeks	RRMM ORR 60%	(159)
				2.5 mg/kg		RRMM ORR 31%	(160)
AMG224	BCMA	Amgen	mAb: IgG1 linker: not cleavable payload: mertansine	Phase I dose-escalation study,	60 min I.V. infusions every 3 weeks	RRMM ORR 23%	(161)
				30–300 mg			
MEDI2228	BCMA	AstraZeneca	mAb: IgG1 linker: valine-alanine protease cleavable payload: tesirine	Phase I dose-escalation study	I.V. infusions every 3 weeks	RRMM ORR 66% at 0.14 mg/kg dose (N = 41)	(162)
				0.0125–0.20 mg/kg			

ADC technologies combine *mAbs* (generally IgG1 due to the availability of multiple lysines required for optimal conjugation), selective for the antigen on the target cell, with *toxic payloads* (generally targeting microtubules or DNA duplication) (164, 165) and a *linker* between the antibody and the cytotoxic agent, as extensively described in excellent reviews (166). Based on ADC design, linkers can be stable in serum or in the circulation, thus after the initial internalization antigen/ADC complex is followed by its complete degradation in the lysosome (166). Otherwise, linkers could be cleaved only under certain specific conditions to ensure drug delivery. For example, the hydrazine linkers are susceptible to acidic conditions, the disulfide linkers to reducing equivalents (glutathione), and the peptide linkers to proteases (164, 165, 167, 168).

The first anti-BCMA mAb *cSG1* was initially evaluated by Seattle Genetics in 2007, developed both as a naked mAb (not further tested in large clinical trials) as well as with a drug conjugate (ADC) (143). Alone, or in combination with bortezomib or lenalidomide, cSG1was able to induce cytotoxicity of myeloma cells *in vitro*, even in the presence of BMSCs, and to reduce the migratory capacity of MM cells through the inhibition of NFkB (120).

The first-in-class anti-BCMA ADC investigated in clinical trials is *Belantamab Mafodotin* (GSK2857916). The *Belantamab Mafodotin* platform has three peculiarities: i) afucosylated IgG1to ensure the highest affinity for the FCγRIIIa/CD16a receptor of the effector cells to mediate ADCC; ii) a protease non-cleavable linker, to avoid serum degradation and release of the payload outside the cell of interest. The linker is cleaved inside the cell; iii) a powerful cytotoxic agent as payload, monomethyl auristatin (MMAF), designed to be much more active when actively delivered inside cells with a mAb, compared to treatment in the untargeted form. Thanks to its peculiar structure, *Belantamab Mafodotin* has different mechanisms of action:

1) induces the arrest of MM cells in G2/M phase resulting in apoptosis
2) induces a powerful ADCC *via* binding of the defucosylated Fc fragment of NK and PBMC cells
3) induces ADCP *via* binding of the defucosylated Fc fragment of macrophages
4) competes with BAFF and APRIL, reducing their signal of activation of NFkB
5) reduces activity of BCMA^+^ dendritic plasmacytoid cells which support proliferation and drug resistance of MM cells (169).

*Belantamab Mafodotin* was first tested in both disseminated and subcutaneous human MM xenograft mouse models where it was shown to induce a complete eradication of the neoplasm without inducing weight loss of the mice, thus confirming the absence of toxicity (170). Subsequently, it was investigated in phase I (171) and phase II trials (160), with encouraging about 30% of overall response in penta-refractory patients and now is being tested in combination with lenalidomide and pomalidomide for patients with relapsed/refractory MM (143), as recently described in several recent comprehensive reviews (172–180). The most common grade 3–4 adverse events include: keratopathy (181, 182), thrombocytopenia, and anemia (160). Blurred vision, keratitis, dry eye, and microcystic epithelial damage are typically associated to ADCs due to o non-specific ADC uptake into actively dividing basal epithelial limbal stem cells residing in the basal epithelial layer of the cornea (183).

Future developments to improve drug-induced toxicities include the combination of Belantamab Mafodotin with both immunomodulatory drugs and proteasome inhibitors, extending dosing intervals (i.e. every 4–6 week dosing versus every 3 week dosing) (179), together with the clinical studies involving other anti-BCMA ADCs with several promising different payloads (184).

Other BCMA-targeting ADCs include AMG-224, CC-99712, SG1-auristatin, MEDI228, and HDP-101, as summarized in very recent comprehensive reviews (172, 185–187).

*AMG 224* is an antihuman BCMA IgG1 antibody conjugated with mertansine, an antitubulin maytansinoid, through a non-cleavable linker. In the dose escalation NCT02561962 phase 1 trial, 40 patients received intravenous AMG 224 every 3 weeks at prespecified doses of 30–300 mg in a 3 + 3 design, with no mandated pre-medications. The objective response rate (ORR) for the study was 23%, including six responses in dose escalation and three responses in the dose expansion. In the dose escalation cohort, the most common AEs include thrombocytopenia, fatigue, nausea, AST increase, and anemia (161).

In *MEDI2228* a fully human BCMA-binding IgG1 antibody is conjugated to DNA-damaging agent pyrrolobenzodiazepine (PBD) *via* a protease-cleavable linker, showing higher clinical activity than to a monomethyl auristatin F (MMAF) analog, also in the presence of high levels of sBCMA, due to induced DNA damage responses (DDR) and synergized with multiple DDR-inhibitors. A phase 1 dose-escalation/-expansion study of MEDI2228 as monotherapy in relapsed/refractory patients is currently ongoing (NCT03489525).

In *HDP-101* a BCMA-specific antibody is conjugated to the RNA polymerase inhibitor amanitin, a synthetic derivative belonging to the amatoxin family, identified more than 40 years ago in the mushroom *Amanita phalloides*. These substances, responsible for severe hepatotoxicity secondary to the ingestion of these fungi, bind with high affinity to RNA Polymerase II, thus reducing transcription and protein synthesis and are effective against both rapidly dividing and resting cells. Based on preclinical studies, HDP-101 has large clinical activity in models with a knockout of tumor suppressor TP53 and knockdown of RNA polymerase POLR2A, which mimics the deletion of 17p in a subtype of high-risk MM patients. Preclinical data have shown that HDP-101 has significant anti-tumor activity both *in vitro* and on xenograft models and results of the clinical study are expected in 2021 (188).

### How to Target BCMA in Multiple Myeloma: The Bispecific Antibodies and Beyond

A further challenge of immunotherapy is retargeting the effector cells (NK-cells, macrophages, T-cells) to provide rapid activation, robust and durable cytotoxic responses, and potentially generate immunologic memory (189). The engagement of CD3 (part of the T-cell receptor) induces both proliferation of CD4 and CD8 T-cells and cytotoxic activity by CD8 and in part CD4 cells against the target. The engagement of CD3 is the major proliferation signal, even though there may be additional indirect mechanism of proliferation induction by cytokines. Thus, the interaction between the patient’s own T lymphocytes and the tumor cells expressing a specific antigen could be facilitated, to eliminate cancer without genetic alteration of the T cells or need for *ex vivo* expansion/manipulation, providing off-the-shelf immuno-oncotherapy (189, 190). In this scenario, the class of bispecific antibodies (bsAbs), also known as dual-targeting molecules, includes antibodies or derived proteins engineered to have multiple binding sites, each with a unique antigen specificity, to different epitopes, to physically bridge two or more cells.

There are two major factors which could affect pharmacokinetics (191) of bsAbs immunotherapy: i) the binding to FcRn (neonatal Fc receptor), which in turn mediates the long half-life of IgG molecules *in vivo* and it is involved in transcytosis from the vascular space out into tissue compartments; ii) the potential higher immunogenicity of anti-drug antibodies due to the presence of non-natural structural motifs (192). To this end, Fc mutations have been heavily employed in new generation mAbs to modify interaction with FC-γ-receptors, to increase or decrease CDC, ADCC, and ADCP. Mutations to modify FcRn binding are also attempted by different groups to modify pharmacokinetics, but these have not reached the clinic (191). ADC hapten-like structure across eight molecules tested in 11 phase I–II clinical trials do not appear to increase patient immune responses beyond those generally observed for mAb biotherapeutics (193), but data lack in MM setting. It is still under investigation if larger molecules could hard penetrate the tumor, especially when extramedullary bulky masses are present.

Two formats of bsAbs have been extensively studied in MM: BiTE (Bispecific T-cell engager, developed by Amgen, Thousand Oaks, CA, USA) (10, 184, 190, 194) and DuoBody (developed by Genmab A/S, Copenhagen, Denmark). In the BiTE molecules, binding domains are two single‐chain variable fragment (scFv) regions, arised from mAbs, joined by a flexible peptide linker: one, to recognize tumor‐expressed antigens, and another to engage effector T-cells. The second scFv binding domain is always specific for CD3, the invariable part of the T‐cell receptor complex. When a BiTE molecule engages both a cytotoxic T cell and a tumor cell, the T cells start to proliferate, increasing overall numbers of effector cells and strengthening the potency of BiTE therapy (195). Once the cytolytic synapse has occurred, the T cells release perforin and granzyme B, thus inducing the apoptosis of the tumor cells. Furthermore, the activation of lymphocytes induces the release of cytokines that amplify the immunological response by involving other immune cells and induce a proliferation of T cells (189, 190, 196).

**Figure 3:**
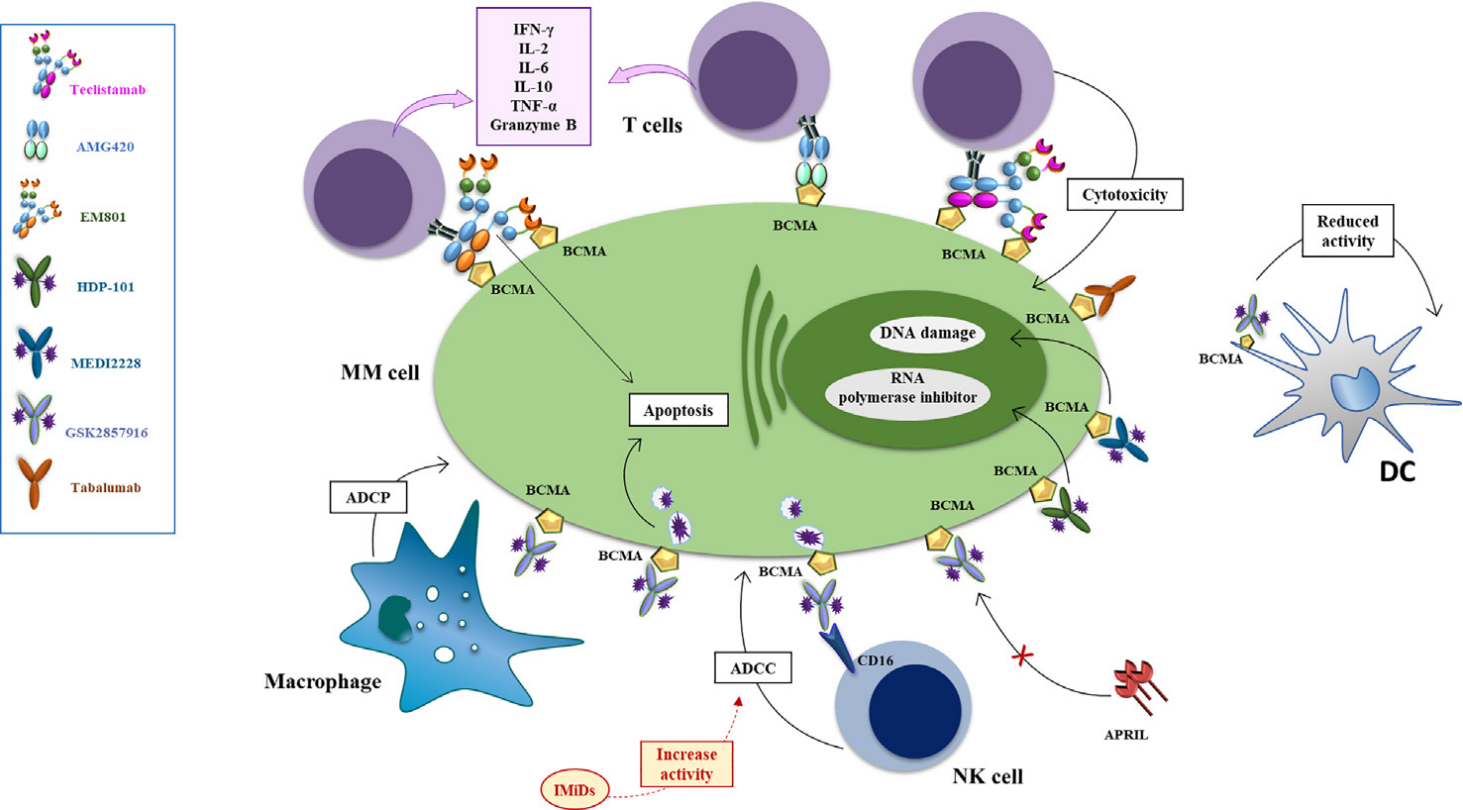
Mechanisms of action of anti-BCMA mAb, antibody drug conjugates and bispecific antibodies. The antibody drug conjugate Belantamab mafodotin exerts anti-MM effect *via* several mechanisms: i. inducing ADCP *via* binding of the defucosylated Fc fragment of macrophages the arrest of MM cells in G2/M phase resulting in apoptosis; ii. inducing a powerful ADCC *via* binding of the defucosylated Fc fragment of NK and PBMC cells (an effect enhanced by combination with lenalidomide) iii. competing with BAFF and APRIL, reducing their signal of activation of NFkB in MM cells (an effect enhanced by combination with bortezomib) iv. reducing activity of BCMA+dendritic plasmacytoid cells which support proliferation and drug resistance of MM cells. Upon binding with MM cells *via* BCMA, MEDI2228 releases pyrrolobenzodiazepine to promote DNA damage and cell death, while HDP-101 releases the RNA polymerase inhibitor amanitin, to reduce transcription and protein synthesis, resulting in apoptosis of both rapidly dividing and resting cells. AMG 224 is an antihuman BCMA IgG1 antibody conjugated with mertansine, to inhibit the assembly of microtubules with consequent cell death. Tabalumab (LY 2127399) is an-anti BAFF human naked monoclonal antibody that neutralizes the membrane-bound and soluble forms of this factor, reducing their signal of activation of NFkB in MM cells. Bispecific monoclonal antibodies can simultaneously bind to two different types of antigen to engage effector cells against neoplastic cells. EM-801 and AMG-420 are two examples of BCMA/CD3 bispecific T-cell engager. Teclistamab is a BCMA/CD3 DuoBody.

*AMG 420 (formerly BI 836909)* was the first anti-BCMA BiTE used in relapsed/refractory MM patients. AMG 420 is a bispecific single-chain variable fragment consisting of two linked single-chain variable fragments (scFvs) (197). The BCMA scFv is positioned N-terminally, and the CD3ϵscFvC-terminally followed by a hexahistidine (His6tag) (197). *In vitro* experiments have documented that both T lymphocyte subpopulations (CD4^+^ and CD8^+^) contribute to the antibody-induced lysis of MM cells, associated to autologous T-cell activation, documented by increased secretion of IFN*γ*, IL-2, IL-6, IL-10, and TNFα in a dose dependent manner, in T-cells obtained from both newly diagnosed and RRMM patients (**Figure 3**). The maximum cytolytic activity was reached between 16 and 24 h, greater in presence of peripheral blood mononuclear cells (PBMCs), suggesting the engagement of other blood cells. The cytolytic activity of AMG420 was not affected by the co-culture of MM cells with stromal cells, that usually confer drug resistance, or in presence of soluble APRIL and BCMA, which could interfere with or bind the antibody. The same encouraging results have been obtained *in vivo*, in both mouse xenograft models with the insertion of human T cells and in cynomolgus monkeys, where a dose-dependent decrease of bone marrow PCs could be documented as well (197). Clinical application of AMG420 was very promising in RRMM setting, with a response rate of 70% at the maximum tolerated dose, including for half patients the achievement of MRD-negative complete response (152). However, further clinical development has been stopped, due to the short half-life of AMG420, requiring a continuous infusion of 4 weeks, and the high rate of infections, mainly due to the requirement of a catheter for i.v. injection.

In the attempt to improve drug manageability and prolong its clearance, new bsAbs have been engineered to have a Fc moiety which makes them more like a complete antibody (198). Among them, *AMG701* has a mean elimination half-life of 112 h (4.7 days) and it is able to induce a potent T-cell-dependent cellular toxicity against BCMA positive MM cell lines, together with a dose-dependent T-cell activation a cytokine secretion. *In vivo*, it was able to inhibit growth of tumor xenografts, prolong survival of an orthotopic mouse xenograft model and deplete MM cells in cynomolgus monkeys (199). In RRMM patients, AMG 701 potently induced autologous cell lysis, T-cell proliferation, and differentiation leading to higher CD8/CD4 ratios, acting synergistically with lenalidomide and pomalidomide to prevent myeloma relapse *in vivo* (200). The interim analysis of the Phase 1 dose escalation trial (NCT03287908) presented at ASH 2020 (153) provided encouraging signs of activity as a single agent in heavily pre-treated MM patients. AMG 701 was given to 85 R/R MM patients who had received at least three prior lines of therapy, and a median of six lines. The response rate was 36% at doses of 3–18 mg with responses lasting up to 26 months in one patient. Six of seven patients, who were tested for minimal residual disease (MRD), were MRD-negative. In the most recent evaluable cohort, there was an 83% ORR, with 4/5 responders being triple refractory (153).

*EM801* is an asymmetric IgG1 bsAb first developed by EngMab AG with Celgene. EM801 incorporates bivalent binding to BCMA, a head-to-tail fusion of BCMA- and CD3ε-binding Fab domains and an engineered Fc region, with completely abolished binding to FcγRs and C1q, carrying a heterodimeric Fc region with intact FcRn binding. The molecular structure enables prolonged half-life to approximately 4 days, thus allowing the possibility of intravenous or subcutaneous administration once a week (201). *In vitro*, EM801 induces a strong, dose dependent bond between T lymphocytes and MM cells with consequent activation of T cells, documented by the hyper-expression of CD25 and CD69 and release of granzyme B and inflammatory cytokines such as IFN*γ*, TNFα, and IL-2 (**Figure 3**). At an E:T ratio of 2:1, 5:1, and 10:1 EM801 induces a killing of MM cells of 60, 70, and 80% respectively and the depletion of a subpopulation of T lymphocytes (CD4^+^ or CD8^+^) not significantly reduces the cytocidal effect of the antibody. Experiments conducted on primary MM cells have documented that EM801 was able to induce mortality in 77 and 83% primary samples obtained respectively from newly diagnosed and RRMM patients, without toxicity on microenvironmental cells. *In vivo*, anticancer activity has been documented in both mouse models and in cynomolgus monkeys, where a single administration induced a significant reduction of BCMA+ cells after only 24 h. A clinical study documented almost a 90% overall response rate, including 44.4% sCR/CR and more than 90% of MRD negativity achievement, with a good toxicity profile (184).

The further development of *CC-93269 (former EM901)*, a human IgG1-based T-cell engager that binds to BCMA and CD3 epsilon in a 2 + 1 format, is promising as well, as shown by Dr. Costa at ASH 2019 (154). In a dose-escalation trial of 30 patients with relapsed or refractory disease, the 10-mg dose of CC-93269 induced responses in 89% of patients, including complete or stringent complete remission in 44%. Minimal residual disease negativity was achieved by 92% of responders. Although cytokine-release syndrome occurred in about three-quarters of patients, cases were mostly grade 1 or 2, and it tapered off after the first dose.

*TNB-383B* and *TNB-384B* have been developed by Tenebio based on *in silico* analysis of heavy chain only/fixed light chain antibody sequences (202). *TNB-383B* is a BCMA x CD3 bispecific T-cell redirecting antibody incorporating an activating a unique anti-CD3 moiety (selective in the -383B platform, pan-T-cell activator in the -384B platform), two heavy-chain-only anti-BCMA moieties for a 2:1 tumor associated antigen to CD3 stoichiometry, and a silenced human IgG4 Fc tail (203). The bivalent BCMA binding reduces APRIL competition, conferring high specificity and avidity to the anti-BCMA moieties. Differently from other pan-T cell activating T-bsAbs that can overstimulate T cells, *TNB-383B* preferentially activates effector over regulatory T-cells and uncouple cytokine release from anti-tumor activity, induce PC lysis, regardless of very high or low E:T ratio (203), reducing the CRS risk, as shown in further clinical development (155, 202, 203). Data presented at last 2020 ASH meeting showed a favorable safety profile in patients with R/R MM and achieved an overall response rate of 80% at doses ≥40 mg every 3 weeks. The most common adverse events were cytokine release syndrome, fatigue, headache, anemia, infection, and nausea. TNB-383B was well tolerated at doses up to 40 mg, without the need for step/split dosing. A preliminary ORR of 52% (12/23) was observed at doses ≥5.4 mg, including deep (6 PR/3 VGPR/3 CR) and durable (up to 24 weeks) responses despite dosing only every 3 weeks (155).

*PF-06863135 (Elranatamab)* is an anti-BCMA x anti-CD3 BsAb that consists of targeting arms within an IgG2a Fc backbone, given with a weekly subcutaneous (SC) infusion to allow higher doses than intravenous administration without increasing adverse events (156, 204). Recent update about its safety and efficacy in RRMM patients has been reported at ASH 2020 by Dr. Lesokhin. Responses were achieved with SC dosing of Elranatamab in 6 of 8 (75%) patients at the two highest dose levels evaluated. However, the enrollment of the phase-2 trial MagnetisMM-3 has been paused in May 2021 in US due to peripheral neuropathy.

Novel mechanisms of drug resistance are emerging, like the loss or reduction of BCMA antigen, requiring alternative antigens to target. A promising antigen is the G-protein-coupled receptor class 5 member D (GPRC5D), expressed selectively at high levels in MM cells and associated to inferior outcome in MM patients, independently from BCMA expression, even if its function and ligand is still largely known (205).

*Talquetamab* (JNJ-64407564) is a GPRC5D x CD3 DuoBody able, *in vitro*, to induce cytotoxicity independently from the number of BCMA receptors or the amount of sBCMA, and, *in vivo*, to recruit T cells at the tumor site, without affecting humoral immunity due to lack of expression on B memory cells. Robust preclinical data provided the rationale for the ongoing phase NCT03399799I clinical trial (122).

*Cevostamab* (BFCR4350A) is a new bispecific antibody developed by Roche that simultaneously binds to the CD3 protein on immune T-cells and a portion of the Fc receptor-like protein 5 (FcRH5), a protein receptor found in nearly all myeloma cells, more highly expressed in cell carrying 1q21 abnormalities (206). Preliminary data discussed at ASH 2020 showed ORR of about 53%, irrespective of target expression level in patients. Deep and durable responses were observed in patients with high-risk cytogenetics, triple-class refractory disease, and/or prior exposure to anti-CD38 monoclonal antibodies, CAR T cells, or antibody-drug conjugates (207). In peripheral blood, Cevostamab induced robust CD8+ T-cell activation and proliferation and IFN-γ induction at active doses (3.6 mg), up to 20-fold higher than at baseline. CD8+ tumor-infiltrating T-cell levels were higher on treatment in responders than in non-responders, and T-cell expansion by end of the first cycle was more pronounced in responders than in non-responders, irrespective of baseline CD8+ T-cell levels (208).

The recent discovery that, under physiological conditions, IgG4 can engage Fab-arms exchange (209), has prompted the technology of DuoBody platform (195, 210, 211). In Duobodies, introducing matched mutations at the CH3 interfaces creates a IgG1 bispecific antibody favoring and then stabilizing Fab arm exchange, for the generation of stable bispecific IgG1 antibodies in which heavy and light chain homodimers from two different antibodies form a single heterodimeric bispecific antibody (211–214).

*Teclistamab* (JNJ-64007957) is a DuoBody bsAb that induces T cell-mediated cytotoxicity against BCMA-expressing myeloma cells, independently from the amount of sBCMA, APRIL, or BAFF (Pillarisetti et al., 2020). *Teclistamab* has shown to be highly active *in vitro* on immortalized and primary myeloma cells, obtained also from daratumumab-refractory patients (215). Teclistamab is currently being evaluated in a Phase 2 clinical study for the treatment of relapsed or refractory multiple myeloma (NCT04557098) and is also being explored in combination studies (NCT04586426, NCT04108195). At ASCO 2020 congress, Dr. Usmani presented excellent preliminary results from the ongoing study of weekly teclistamab in RRMM (NCT03145181), with manageable safety across all doses explored and 78% overall response rate (102). However, the efficacy of Teclistamab was inversely related to the PDL-1 expression on RRMM cells, thus ongoing trials are investigating the possibility of overcoming this resistance using a combination with PDL1 inhibitors (216). Additional partners for combination therapy include γ-secretase inhibitors which potentiate Teclistamab killing capacity by elevating BCMA surface expression (215).

*HPN217* is a tri-specific T cell activating construct (TriTAC) consisting of three binding domains: an N-terminal single domain antibody (sdAb) that binds to human BCMA, a middle sdAb that binds to human serum albumin (HSA), and a C-terminal single chain Fv (scFv) that binds to CD3ϵ of the T cell receptor (TCR) complex (217). The *in vitro* pharmacological activity of HPN217 was evaluated by T cell-dependent cellular cytotoxicity (TDCC) assays. In co-cultures of T cells from normal human or cynomolgus monkey donors, target tumor cells, and HSA, HPN217 mediated dose-dependent and BCMA-dependent cytotoxicity with EC50 values ranging from 0.05 to 0.7 nM. Killing was dependent on expression of BCMA on target tumor cells.

Non-clinical *in vivo* properties of HPN217 were evaluated in xenograft models and a single dose pharmacokinetic (PK) study in cynomolgus monkeys. HPN217 mediated dose-dependent growth suppression against the RPMI-8226 MM model and Jeko-1 mantle cell lymphoma model expressing relatively low levels of 5,600 and 2,200 copies of BCMA per cell, respectively. Serum half-life, volume of distribution, and clearance appeared to be independent of dose. HPN217 was demonstrated to be stable and remained intact up to 3 weeks *in vivo* as demonstrated by a functional ligand binding assay using recombinant CD3ϵ and BCMA, respectively, to capture and detect HPN217. Importantly, serum samples collected 1 week after dosing were as potent as stock HPN217 in MM tumor cell killing in TDCC assays.

*RO7297089* is a potent therapeutic agent *in vitro* and selectively kills BCMA expressing MM-PCs by activating innate immunity, *via* ADCC and ADCP, with low incidence of acute cytokine release. In a 1-month repeat-dose study in cynomolgus monkeys, RO7297089 was well tolerated, and there were no test article-related adverse effects at up to 50 mg/kg, with no significant cytokine release. RO7297089 represents a novel and promising MOA with a favorable safety profile, distinct from the T cell-based BCMA-targeting modalities in the clinic (218, 219).

*CTX‐4419*, is a first‐in‐class NKp30xBCMA bispecific, able to induce cytokine production, NK-cell proliferation, and potent tumor cell killing of target cells, independently from high, intermediate, or low BCMA expression. Differently BCMA‐IgG1 mAbs can activate NK cells in the absence of CD16A engagement (220).

*2A9-MICA* consists of human MICA extracellular region and a single-chain antibody fragment (scFv) that targets BCMA generated by phage display technology. *In vitro*, 2A9-MICA activated NK cell-mediated cytotoxicity and induced NK cells to kill BCMA-positive human myeloma cells. Moreover, in BCMA-positive, MM-bearing nude mice, 2A9-MICA specifically targeted tumor tissue, where it effectively recruited immune cells and inhibited tumor tissue growth showed superior antitumor activity (221).

## Blocking PD-1/PDl-1 Axis by mAbs in MM

Several experimental pre-clinical data indicate that PD-L1/PD-1 blockade by mAbs provided promising anti-MM effects. *In vitro* PD-L1/PD-1 blockade overcame BM MSC-mediated MM growth and directly enhanced NK and T cell mediated anti-MM responses (222, 223). MM cells by PD-L1 expression inhibit the activity of CTLs, acquiring a proliferative advantage which results in immune evasion and resistance to anti-myeloma drugs (224). *In vivo* PD-L1 blockade prolonged mice survival after stem-cell transplantation (225–228) as well as PD-1 blockade also prolonged the survival in disseminated myeloma-bearing mice (228, 229), by mainly acting on CD4^+^ or CD8^+^ T cells (229). In these models, PD-1 expression on both CD8^+^ and CD4^+^ T cells was higher in mice with advanced MM as compared to non-tumor bearing ones.

mAbs targeting the PD-1/PD-L1 axis can be divided into two different groups: (i) those against the PD-1 receptor and (ii) those against the ligands (PD-L1/PD-L2). Nivolumab, pembrolizumab, and pidilizumab are the main anti-PD-1 mAbs used whereas anti-PD-L1 mAbs are durvalumab and atezolizumab. However, despite promising pre-clinical data, the use of mAbs antiPD-1/PD-L1 mAbs as single agents did not show a significant clinical effect in relapsed refractory MM. On the other hand, the phase III trial evaluating lenalidomide and dexamethasone in combination with pembrolizumab in patients with MM presented unexpected safety findings and was discontinued. Accordingly, the other clinical trials anti PD-1/PD-L1 mAbs in combination with IMiDs have been interrupted. Actually the identification of the best MM patients candidate to the treatment with PD-1/PD-L1 blockade is still be unknown.

## Conclusions: Challenges and Perspectives of Immunotherapy in Multiple Myeloma

Immunotherapy is revolutionizing the therapeutic scenario of both newly diagnosed and refractory-relapsed MM patients. Novel challenges are emerging on how to choose the target and the therapeutic format as summarized in **Table 5**, the best sequential approach, timing, and patients’ characteristics.

**Table 5:** Vantages and Disadvantage of monoclonal antibodies, Bispecific antibodies, Antibody drug conjugated and CAR-T cells.

Target	Therapeutic format	Advantage	Disadvantage
**CD38**	Naked monoclonal antibody	High clinical activity in triplets and quadruplets (dara-based regimens are novel standard of care for elderly patients).	Reduction of CD38+ activated T-cells. Perturbation of T-cell compartment.
		The target is generally unaffected by disease stage	
	Bispecific antibody	No lymphodepletion regimen required No delay in treatment because they are “off the shell” products	Neurotoxicity, cytokine release syndrome (CRS) Short half-life and they need continuous infusion
**SLAMF7**	Naked monoclonal antibody (elotuzumab)	The target is slightly reduced during disease progression. However, SLAMF7 expression is retained in MM patients with relapsed/refractory disease, and after intensive prior therapy.	Lack of relevant clinical efficacy of elotuzumab as single agent or in triplets given frontline; it requires to be part of combination regimens
	Bispecific antibody	T-cell mediated cytolysis independent of major histocompatibility complex.	Short half-life and they need continuous infusion Multiple dosing is expected to elicit a durable response, with intermittent infusions (usually every 3 weeks)
	CAR-T cells	A virus-free CAR gene transfer using advanced Sleeping Beauty (SB) transposon technology. SB transposition in CAR-T engineering is attractive due to the high rate of stable CAR gene transfer enabled by optimized hyperactive SB100X transposase and transposon combinations, encoded by mRNA and minicircle DNA, respectively, as preferred vector embodiments (CARAMBA PROJECT). The allogenic anti-SLAMF7-CAR T cell (UCARTCS1) is the first ‘off-the-shelf’ CAR T-cell product in MM	Restrictive eligibility criteria (adequate heart, liver, and kidney function) SB technology requires lower biosafety level translating to lower infrastructure costs for manufacturing and quality control and high modularity
**BCMA**	Antibody drug conjugated	Off-the-shelf products, immediately available for patients with aggressive disease	Toxicity due to linker-payloads constructs (keratopathy for ADCs using anti mitotic agents). Potential lower response rate as single agents. Multiple dosing is expected to elicit a durable response, with intermittent infusions (usually every 3 weeks)
		Action independent from autologous T-cell fitness and host immune function (ideal for elderly patients).	
	Bispecific antibody	Off-the-shelf products, immediately available for patients with aggressive disease Limited CRS (AMG420), extended half-life from dosing once a week (AMG701, CC-93269) to every 3 weeks (TNB-383B). Subcutaneous administration is intended to allow higher doses than intravenous administration without increasing adverse events and limited CRS (PF-06863135).	Cytokine release syndrome (CRS) Immune effector cells associated neurotoxicity syndrome (ICANS) Higher doses required for antigen target modulation AMG420: continuous I.V. infusion limits the patients’ compliance and quality of life, increased risk of catheter-related infections, neurological toxicity. PF-06863135: polyneuropathy Short half-life and they need continuous infusion
			Mechanisms of resistance: antigen loss or downregulation; immune response against BsAbs constructs; interference with sBCMA
	CAR-T cells	Usually only one infusion is needed The most potent single agent available in the RRMM setting CRS and neuropathy are usually grade 1–2 and manageable	Logistical challenges: lag time because of manufacturing Lymphodepleting conditioning chemotherapy required Cytopenias (sometimes severe and persistent) Limited persistence given the dependence on autologous T-cell fitness and host immune function Short-term remission duration Requirement of defined T-cell subset compositions and humanized targeting domains to reduce immunogenicity and promote engraftment and in *vivo* expansion High costs Exhaustion of manufacturing capacities of centralized and highly specialized GMP production facilities

CAR-T cell therapy against BCMA is one of the most powerful single-agent for RRMM patients (with ORR range 50–90% across the studies), but it is affected by logistical constrains, with up to 20% drop-out rate in the manufacturing time (4–7 weeks) for complications associated with disease progression (230). To overcome these limitations, reduce the high costs and face with exhaustion of manufacturing capacities of centralized and highly specialized production facilities, some technological improvements are ongoing, including virus-free gene transfer, automated point-of-care production and allogeneic cell products to provide off-the-shelf CAR-T cells products (113, 231, 232).

The limited persistence and lack of survival plateau is an additional limitation of CAR-T cells in MM. Some authors suggest to identify upfront (e.g. high risk patients with extramedullary presentation or adverse cytogenetics or biallelic TP53 inactivation) or early during the treatment (e.g. after induction or at first minimal residual disease detection after autologous stem cell transplantation) those patients with the highest chance of benefiting from T-cells redirecting therapies with the final goal of cure (231) or achievement of persistence of minimal residual disease negativity (233). However, T-cell function looks like compromised since the asymptomatic phase of disease (234–236), arising the question about how to improve the efficacy of current immunotherapy approaches and their toxicity profile. For example, T cell proliferation decreased in presence of mature neutrophils (234, 236–238). The cytotoxic potential of T cells engaged by EM801 increased notably with the depletion of mature neutrophils (236), arising the question if immunotherapy should be adapted to an extensive immune profiling not limited to T-cells only.

In immunotherapy of both solid and hematological cancers, there is an increasing evidence about the prominent role of antibody’s constant region, much of which is mediated through interaction of the Fc with FcγRs, that could be engineered to modify their pharmacokinetics and pharmacodynamics. Neutrophils could positively affect the activity of several mAbs, *in vitro* and *in vivo*, *via* the recognition of IgA‐opsonized tumor targets by FcαRI/CD64, as recently reviewed (239). However, the mechanisms of neutrophil‐induced tumor killing are still under debate and the role of neutrophils, either positive or negative, is far from clear.

Reducing progressively the costs, potential toxicity, and therapy complexity is the major strength of off-the-shelf immunotherapeutic strategies like bispecific antibodies and ADCs, which function is largely independent from autologous T-cell fitness and host immune function. Cytokine release syndrome and immune effector cells associated neurotoxicity syndrome can be managed at lower dosages or changing the format to reduce non-specific T-cell activation (as in TNB-383B).

While clinical trials are not yet mature for a direct comparison of several classes of agents and we still need larger series and further confirmation, the favorable safety profile of the first-in-class belantamab mafodotin, makes it a potential great choice for the elderly patients (232), especially when the therapeutic goal is not disease eradication but long-term control of disease, with repeated infusions. The most relevant challenge for the ADCs development is to reduce toxicity related to linker-payloads constructs, like keratopathy associated to ADCs with anti-mitotic agents and neuropathy (240).

The mechanisms involved in the acquired resistance to anti-CD38 mAbs are not fully understood but could involve the downregulation of the CD38 on cell surface, NK and T cells number and exhaustion, overexpression of complement inhibitory proteins, and expression of inhibitory pathways as CD47-SIRPα (45, 49). The therapy in combination with immunomodulatory drugs seems to potentiate the effect of the anti-CD38 mAbs compared with single-agent treatment, increasing the activity of NK cells and on macrophage (9). These mechanisms lead to reach significant response even in relapsed/refractory MM patients. Other drugs (HDACs and ATRA) could overcome the resistance to anti-CD38 mAbs increasing the expression of the CD38 molecule (28, 72, 73).

In contrast to BCMA, CD38 and SLAMF7 antigens show stable expression levels throughout the successive lines of MM treatments, making their contemporary dual targeting an emerging therapeutic option. Hemibodies are a pair of complementary antibody fragments that redirect T cells against cancer-defining antigen combinations. Hemibodies addressing CD38 and SLAMF7 recruit T cells for the exquisite elimination of dual antigen positive multiple myeloma cells while leaving single antigen positive bystanders unharmed. Differently from T-cell redirecting therapies targeting only CD38 and SLAMF7 targeting hemibodies do not induce massive cytokine release and T cell fratricide reactions, translating into very low off-tumor toxicity in clinical settings (241).

The unique mechanisms of action of monoclonal antibodies make them a perfect component to be used alone or in combination with present therapeutic treatments, which could improve the efficacy of the treatment and probably overcome resistance (114). In the upcoming years, a robust selection of patients, based on both genomic and immune profiling to test respectively the clonal architecture and the host immune fitness, combined to multi-target immunotherapy could induce a further major paradigm shift to offer long-term control of disease and hopefully cure to most of MM patients.

## Author Contributions

Conceptualization: FR and NG. PS and AR, writing the paper; FR and NG—writing and editing. VM prepared the figures. LN and GS prepared the tables. LC revised the manuscript. All authors contributed to the article and approved the submitted version.

## Funding

This work was supported in part by two grants from the Associazione Italiana per la Ricerca sul Cancro (AIRC) IG2017 n. 20299 (NG), IG n.22131 (FR), Ministero della Salute Italiana PE-2016-02361261 and Università degli Studi di Catania, “Fondi di ateneo 2020-2022, Università di Catania, linea Open Access.”

## Conflict of Interest

NG received research funding and honoraria from Amgen, Bristol Mayers Squibb, Celgene, Millenium Pharmaceutical, and Janssen Pharmaceutical. AR and FR received research funding and honoraria from Amgen.

The remaining authors declare that the research was conducted in the absence of any commercial or financial relationships that could be construed as a potential conflict of interest.
